# Management of acute coronary syndrome in South Africa: insights from the ACCESS (Acute Coronary Events – a Multinational Survey of Current Management Strategies) registry

**DOI:** 10.5830/CVJA-2012-017

**Published:** 2012-08

**Authors:** Colin Schamroth

**Affiliations:** Milpark Hospital, Johannesburg, South Africa

**Keywords:** acute coronary syndrome, myocardial infarction, unstable angina, registry, death

## Abstract

**Background:**

The burden of cardiovascular diseases is predicted to escalate in developing countries. While many studies have reported the descriptive epidemiology, practice patterns and outcomes of patients hospitalised with acute coronary syndromes (ACS), these have largely been confined to the developed nations.

**Methods:**

In this prospective, observational registry, 12 068 adults hospitalised with a diagnosis of ACS were enrolled between January 2007 and January 2008 at 134 sites in 19 countries in Africa, Latin America and the Middle East. Data on patient characteristics, treatment and outcomes were collected.

**Results:**

Of the 642 patients from South Africa in the registry, 615 had a confirmed ACS diagnosis and form the basis of this report; 41% had a discharge diagnosis of ST-segment elevation myocardial infarction (STEMI) and 59% a diagnosis of non-ST-segment elevation acute coronary syndrome (NSTE-ACS), including 32% with non-ST-segment elevation myocardial infarction (NSTEMI) and 27% with unstable angina (UA).

During hospitalisation, most patients received aspirin (94%) and a lipid-lowering medication (91%); 69% received a beta-blocker, and 66% an ACE inhibitor/angiotensin receptor blocker. Thrombolytic therapy was used in only 18% of subjects (36% of STEMI patients and 5.5% of NSTE-ACS patients). Angiography was undertaken in 93% of patients (61.3% on the first day), of whom 53% had a percutaneous coronary intervention (PCI) and 14% were referred for coronary artery bypass surgery. Drug-eluting stents were used in 57.9% of cases. Clopidogrel was prescribed at discharge from hospital in 62.2% of patients.

All-cause death at 12 months was 5.7%, and was higher in patients with STEMI versus non-ST-elevation ACS (6.7 vs 5.0%, *p* < 0.0001). Clinical factors associated with higher risk of death at 12 months included age ≥ 70 years, presence of diabetes mellitus on admission, and a history of stroke/transient ischaemic attack (TIA).

**Conclusions:**

In this observational study of ACS patients, the use of evidence-based pharmacological therapies for ACS was quite high. Interventional rates were high compared to international standards, and in particular the use of drug-eluting stents, yet the clinical outcomes (mortality, re-admission rates and severe bleeding episodes at one year) were favourable, with low rates compared with other studies.

## Abstract

Knowledge on the prevention and treatment of cardiovascular diseases derives from randomised, controlled clinical trials and observational studies. Registries of treatment patterns of particular disease processes have shaped and influenced treatment practices. These observational studies have also helped map out differences in the populations studied, whether this be geographical or ethnic.

Current data derive almost exclusively from the developed world populations, and whether these data are applicable to population groups outside of the developed world is unknown.^1^-^6^ Knowledge on treatment trends and practices from the developing world is scanty or entirely absent. While studies such as the INTERHEART have demonstrated that the risk factors for the development of acute myocardial infarction are similar across population groups, the patterns of treatment of ischaemic heart disease among different population groups in underdeveloped nations remains unknown.^7^

Cardiovascular disease mortality and associated major risk factors (elevated blood pressure, raised cholesterol levels, cigarette smoking, diabetes mellitus, physical inactivity and high-fat diet) vary widely from country to country, but it is predicted that within the next decade, the major burden of cardiovascular diseases will shift to the developing countries.^8^ There is therefore a need to establish registries in developing countries to increase awareness of the cardiovascular disease burden and establish appropriate preventive and management strategies. To date there are no published registries on the management and outcomes of patients admitted to hospital for acute coronary syndromes in South Africa.

The aim of the multinational observational ACCESS (Acute Coronary Events – a Multinational Survey of Current Management Strategies) registry was to gain insights into the descriptive epidemiology, current practice patterns, and one-year outcomes of patients hospitalised with acute coronary syndrome (ACS), whether this be unstable angina (UA)/non-ST-segment elevation acute coronary syndrome (NSTE-ACS) or ST-segment elevation myocardial infarction (STEMI), in developing countries in Africa, Latin America and the Middle East, excluding countries from Europe and North America.

The complete ACCESS study design, methods and results have previously been published.^9^ The following report is of the data applicable to the South African patient cohort, and how these compare with the data from the ACCESS study as a whole.

## Methods

ACCESS was a prospective, observational, multinational registry in patients hospitalised for an acute coronary event. Patients were enrolled at 134 sites in 19 countries in North Africa (Algeria, Morocco, Tunisia), South Africa, Latin America (Argentina, Brazil, Colombia, Dominican Republic, Ecuador, Guatemala, Mexico, Venezuela) and the Middle East (Egypt, Iran, Jordan, Kuwait, Lebanon, Saudi Arabia, United Arab Emirates).

The ACCESS registry was conducted in accordance with the Guidelines for Good Clinical Practice and under the leadership of a scientific advisory board (the ACCESS steering committee). Each participating country had the responsibility of ensuring locally that all necessary regulatory submissions were performed in accordance with local regulations, including data-protection regulations. Local institutional review boards or independent ethics committees approved the study and all patients provided informed consent to participate.

Selection of physicians was determined at the country level; 29 sites participated in South Africa. The aim was to enroll approximately 25 patients per site.

Patients aged 21 years and older who were admitted alive to hospital with an ACS and provided signed, informed consent were eligible to participate in this all-comers registry. Patients with symptoms precipitated by a secondary co-morbidity (e.g. anaemia, heart failure or non-cardiac trauma) and patients participating in concomitant clinical trials were excluded from the study. Eligible patients who agreed to participate were recruited consecutively to avoid patient selection bias.

To be enrolled, patients had to present with ischaemic symptoms of ACS within 24 hours of hospital presentation and have at least one of the following: (1) electrocardiographic changes [transient ST-segment elevation ≥ 1 mm, ST-segment depression ≥ 1 mm, new T-wave inversion ≥ 1 mm, pseudo-normalisation of previously inverted T waves, new Q waves (one-third of the height of the R wave or > 0.04 s), new R wave > S wave in lead V1, or new left bundle branch block]; (2) documentation of coronary artery disease [history of myocardial infarction (MI), angina, congestive heart failure believed to be due to ischaemia or resuscitated sudden cardiac death, history of or new positive stress test with imaging, previous or new cardiac catheterisation documenting coronary artery disease, prior or new percutaneous coronary intervention (PCI) or coronary artery bypass surgery (CABG)]; and (3) an increase in a cardiac biochemical marker of myocardial necrosis (troponin or CK-MB).

The data were recorded prospectively on admission to hospital (baseline), at discharge, and at 6 ± 1 months’ and 12 ± 1 months’ follow-up visits. Data were collected via telephonic interviews for patients who did not attend the follow-up appointments.

In each country, 10% of sites that enrolled at least one patient were randomly selected to undergo a site visit and data audit. At each of these sites, 100% of case report forms for all enrolled patients were monitored for source documentation and accuracy.

The primary endpoint was all-cause death at one year from initial hospitalisation. Secondary endpoints (one year from initial hospitalisation) included cardiovascular death, cardiovascular death and non-fatal MI, non-fatal stroke, non-fatal MI, the combined endpoint of cardiovascular death, stroke or myocardial infarction and re-hospitalisation for ischaemic events, the composite endpoint in each country, and bleeding episodes. All-cause death at 30 days was also recorded.

## Results

Recruitment of patients took place between January 2007 and January 2008. Of the 39 participating doctors in the 29 South African recruiting sites, 26 identified themselves as cardiologists, of whom 10 (38.5%) were interventional cardiologists. All major geographic areas in South Africa were involved in recruitment of registry patients. A list of the participating doctors is given at the end of the article.

**Fig. 1.**
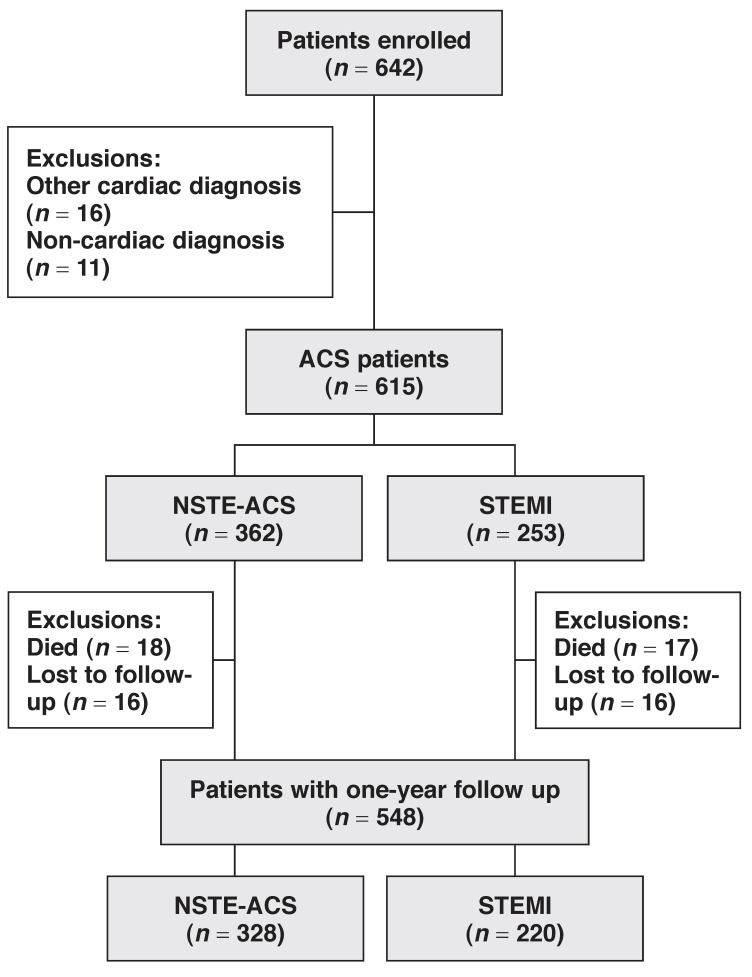
Study flow chart.

A total of 642 patients were enrolled in South Africa, 5.3% of the 12 068 enrolled in the entire ACCESS registry. In the total ACCESS study, 11 731 patients were confirmed as having an ACS. A flow chart of the study patients is given in Fig. 1. In South Africa, of the 642 total patients, 615 patients were confirmed as having an ACS and form the basis of this report. Completed data at the one-year follow up was available on 548 (89.1%) patients; 35(5.7%) had died and 32 (5.2%) were lost to follow up.

Diagnosis at discharge (end of hospital stay) was STEMI in 41.1% and NSTE-ACS in 58.9%, including non-ST-segment elevation MI in 32.4% and unstable angina (no biomarker elevation) in 26.4%. Four patients enrolled in the survey had a ‘discharge’ diagnosis of death, indicating a 0.65% in-hospital mortality rate. In the entire ACCESS study, overall the in-hospital death rate was 2.6%.

Within the South African cohort, 60.7% were Caucasian, 22.8% Asian, 10.7% of mixed ancestry and 5.9% of African ancestry. The population was predominantly male (75.9%) with a mean age of 58.0 ± 12.1 years. STEMI patients were younger than NSTE-ACS patients (mean age 54.5 vs 60.5 years).

Selected patient baseline characteristics are shown in Table 1; 46.7% of patients had a history of angina, 29% a previous MI, and 6% a history of either cerebro- or peripheral vascular disease. Fifty-two per cent of patients gave a family history of cardiovascular disease, far higher than the 31% of the entire ACCESS cohort, and more patients had dyslipidaemia (56 vs 40% for the entire ACCESS population). More patients had had a prior CABG (10 vs 5%), previous PCI (19 vs 12%) and positive current smoking status (44 vs 39%). However there were fewer patients with pre-existing diabetes mellitus (24 vs 35%), but equal numbers of hypertensive subjects (56 vs 55%). Diabetic patients tended to be poorly controlled with a glycosylated haemoglobin (HbA_1c_) level on admission of > 8.0% in 37.3% of patients. Diabetic patients with STEMI were more likely to have an HbA_1c_ level > 8.0% than those with NSTE-ACS (46.7 vs 31.1%).

**Table 1.** Patient Baseline Characteristics: South African Cohort According To Discharge Diagnosis And Overall, And Complete Access Study Overall

	*South Africa*			*ACCESS*
*Discharge diagnosis*	*NSTE-ACS (n = 362)*	*STEMI (n = 253)*	*All (n = 615)*	*All (n = 12 068)*
Myocardial infarction	119 (32.9)	62 (24.5)	181 (29.4)	2588 (21.4)
Stroke/TIA	6 (1.7)	4 (1.6)	10 (1.6)	493 (4)
Angina	216 (59.7)	71 (28.1)	287 (46.7)	(43.8)
Family history of cardiovascular disease	194 (53.6)	126 (49.8)	320 (52)	3728 (30.8)
Congestive heart failure	27 (7.5)	9 (3.6)	36 (5.9)	640 (5.3)
Peripheral arterial disease	18 (5)	8 (3.2)	26 (4.2)	541 (4.4)
Prior PCI	85 (23.5)	32 (12.6)	117 (19)	1483 (12.2)
Prior PCI: Stent	75 (88.2)	26 (81.3)	101 (86.3)	N/A
no stent	10 (11.8)	6 (18.7)	16 (13.7)	N/A
Prior CABG	57 (15.7)	6 (2.4)	63 (10.2)	602 (4.9)
Diabetes	97 (26.8)	50 (19.8)	147 (23.9)	4208 (34.8)
Hypertension	238 (65.7)	104 (41.1)	342 (55.6)	6655 (55.1)
Dyslipidaemia	228 (63.0)	117 (46.2)	345 (56.1)	4864 (40.3)
Current smoking	133 (37.0)	135 (53.6)	268 (43.9)	4730 (39.1)
Body mass index (kg/m^2^)	28.7	28.1	28.4	27.0

PCI: percutaneous coronary intervention; CABG: coronary artery bypass graft. Unless otherwise indicated, results are reported as number (percent); ACCESS includes South Africa.

Forty-nine per cent of patients admitted either moderate or heavy alcohol consumption. Patients were predominantly overweight [body mass index (BMI) 28.4 ± 5.2 kg/m^2^], with only 25% of patients having a BMI below 25 kg/m^2^. Women with STEMI had a larger waist circumference than the women with NSTE-ACS (98.6 ± 13.6 vs 95.1 ± 14.0 cm), while men with STEMI had smaller waist circumferences than those presenting with NSTE-ACS (99.9 ± 12.6 cm vs 103.1 ± 12.5 cm).

Patients presenting with STEMI arrived at hospital sooner after onset of symptoms than the NSTE-ACS patients [median (Q1; Q3) 3.6 (1.6; 9.5) vs 7.4 (2.5; 25.7) hours], with 64.4% of STEMI patients arriving within six hours and 85.3% of the group within 12 hours of the onset of symptoms. This was despite 63.8% of patients living within a 30-minute drive from the admitting hospital. In the complete ACCESS database, the median delay from onset of symptoms to arrival in hospital was 4.0 (1.8; 12.6) hours for STEMI patients and 6.0 (2.0; 19.0) hours for NSTE-ACS patients.

Patients with a discharge diagnosis of STEMI were more likely to have ischaemic symptoms (91.7 vs 69.6%), ECG changes suggestive of ischaemia (97.2 vs 59.7%) and elevated cardiac biomarkers (67.2 vs 53.3%) than patients with a discharge diagnosis of NSTE-ACS. Left bundle branch block was noted in 2.9% of patients overall (3.9% of NSTE-ACS and 1.6% of STEMI).

Prior to hospitalisation, treatment with aspirin was commenced in 43.1% of patients, clopidogrel in 1.6%, unfractionated heparin (UFH) in 2.3% and low-molecular weight heparin (LMWH) in 1.5%. No form of thrombolytic therapy was administered pre-hospitalisation.

Selected interventions and in-hospital treatments are shown in Table 2. Only 18% of patients received a thrombolytic on admission (36.0% of the STEMI patients and 5.5% of the NSTE-ACS patients). Less than 1% of patients were considered to have a contraindication to the use of a thrombolytic agent. Streptokinase was the thrombolytic agent of choice in 54.5%, TNK-tPA in 30.3%, t-PA in 10.1% and r-PA in 5.1% of patients. The thrombolytic therapy was administered in association with LMWH in 36% and UFH in 26% of subjects. In 11% of patients, glycoprotein IIb/IIIa inhibitors were given on the same day as the thrombolytic therapy. This may reflect usage due to failed reperfusion and patients being referred for urgent rescue PCI.

**Table 2.** Selected In-Hospital Interventions And Drug Treatments: South African Cohort According To Discharge Diagnosis And Overall, And Complete Access Study Overall

	*South Africa*			*ACCESS*
*In-hospital interventions and drug treatments*	*NSTE-ACS (n = 362)*	*STEMI (n = 253)*	*All (n = 615)*	*All (n = 11731)*
Thrombolytics	20 (5.5)	91 (36)	111 (18)	2127 (18.1)
Angiography	344 (95)	228 (90.1)	572 (93)	6787 (57.8)
PCI	179 (49.4)	151 (59.7)	330 (53.7)	4141 (35.2)
CABG	70 (19.3)	20 (7.9)	90 (14.6)	668 (5.6)
Stent (% of PCI)	172 (96.1)	139 (92.1)	311 (94.2)	3900 (33.2)
Drug-eluting stent (% of total stent usage)	108 (62.8)	72 (51.8)	180 (57.9)	1713 (43.9)
Aspirin	331 (91.4)	247 (97.6)	578 (94)	10920 (93)
Unfractionated heparin	131 (36.2)	113 (44.7)	244 (39.7)	4636 (39.5)
LMWH	264 (72.9)	188 (74.3)	452 (73.5)	(7144) (60.8)

PCI: percutaneous coronary intervention; CABG: coronary artery bypass graft; LMWH: low-molecular weight heparin. Unless otherwise indicated, results are reported as number (percent); ACCESS includes South Africa.

Overall, 93% of patients underwent angiography (90.1% of STEMI and 95.0% of NSTE-ACS patients). Fifty-three per cent went on to have PCI (59.7% of STEMI and 49.4% of NSTEACS patients), while 14.6% were referred for CABG (7.9% of STEMI and 19.3% of NSTE-ACS subjects). Of the patients referred for PCI, 94.2% had at least one stent inserted (92.1% of STEMI and 96.1% of NSTE-ACS patients), and in 57.9% of cases (51.8% of STEMI and 62.8% of NSTE-ACS patients), this was a drug-eluting stent. PCI was performed within 24 hours of hospitalisation in 61.3% of cases. There were 88 patients (34.8%) who received thrombolytic therapy but who did not exhibit reperfusion and were taken for PCI on the first day of hospitalisation.

Most patients (94% of STEMI and NSTE-ACS subjects) received aspirin, 29.3% received a glycoprotein IIb/IIIa inhibitor, 58.7% a thienopyridine loading dose, and 65.9% a thienopyridine maintenance dose. All thienopyridine use was clopidogrel. UFH heparin was used in 39.7% of patients, and LMWH in 63.4%. The LMWH was almost exclusively enoxaparin. During hospitalisation, 560 (91.1%) patients received a statin, 13.2% a calcium channel blocker (CCB), 69.9% a beta-blocker (BB), 61.1% an angiotensin converting enzyme inhibitor (ACEI) and 5.2% an angiotensin II receptor blocker (ARB). In-hospital bleeding was reported in 21 patients (3.4%); 12 (4.7%) with STEMI and nine (2.5%) with NSTE-ACS.

## Post-hospital management and follow up

Ninety-two per cent of patients were discharged from hospital on aspirin, 62.2% on clopidogrel, 93.3% on a statin, 13.6% on a CCB, 67.4% on a beta-blocker, 61.4% on an ACE inhibitor, and 5.9% on an ARB (Table 3). The rates of use of these drugs were higher among patients with STEMI than in those with NSTE-ACS, with the exception of the use of ARBs.

**Table 3.** Selected Drug Treatments At Discharge: South African Cohort According To Discharge Diagnosis And Overall, And Complete Access Study Overall

	*South Africa*			*ACCESS*
*Discharge drug treatments*	*NSTE-ACS (n = 360)*	*STEMI (n = 251)*	*All (n = 611)*	*All (n = 11 427)*
Aspirin	333 (92.5)	233 (92.8)	566 (92.6)	90.1
Thienopyridine	208 (57.8)	172 (68.5)	380 (62.2)	76.1
Statin	330 (91.7)	240 (95.6)	570 (93.3)	89.2
Calcium channel blockers	65 (18.1)	18 (7.2)	83 (13.6)	16.5
Beta-blocker	222 (61.7)	190 (75.7)	412 (67.4)	75.8
ACE inhibitor	195 (54.2)	180 (71.7)	375 (61.4)	64.3
ARB	26 (7.2)	10 (4)	36 (5.9)	11.3

ARB: angiotensin receptor blocker Unless otherwise indicated result are reported as number (percent); ACCESS includes South Africa.

Follow-up information was obtained telephonically in 58% of cases. At 12 months, 91 (15.6%) patients had had at least one further cardiac-related hospitalisation (41 for STEMI and 50 for NSTE-ACS patients). Unstable angina (i.e. no cardiac biomarker elevation) was the reason for re-hospitalisation in 49 (8.6%) cases and occurred 166 days (mean) after the index hospitalisation. Only four patients were re-admitted for NSTE-ACS and six for STEMI, 253 and 116 days (mean) after the index hospitalisation, respectively. Six patients reported stroke or TIA following hospitalisation and there were 14 re-admissions for heart failure. Bleeding episodes after discharge from hospital for the primary event occurred in 10 (1.8%) of the subjects, five (2.2%) in STEMI and five (1.5%) in NSTE-ACS patients.

At one year, 80.2% of patients were still taking aspirin, 77.3% statin,19% clopidogrel, 10.3% CCB, 53.8% beta-blockers, 46% ACE inhibitors, and 3.8% ARB.

Thirty-day death rates were 2.4% for STEMI and 1.7% for NSTE-ACS patients. Thirty-five patients (5.7%) had died by one year (± 35 days). The one-year mortality rate for patients admitted for STEMI was 6.7% and for NSTE-ACS, 5.0%. Causes of death were fatal MI (eight patients), fatal stroke (two), other cardiovascular including sudden death (12), non-cardiovascular (six), and unknown (seven). Predictors of the primary endpoint outcome (all-cause death at one year) were age ≥ 70 years (*p* = 0.0049), history of stroke/TIA (*p* = 0.0396), and diabetes (*p* = 0.0439).

## Discussion

This registry is the first in South Africa to document the demographics and management strategies used in patients admitted to hospital with a diagnosis of acute coronary syndrome. Patients with pre-existing risk factors of hypertension, diabetes and/or dyslipidaemia were more likely to present with NSTE-ACS, while smokers were more likely to present with STEMI. STEMI patients were also younger than NSTE-ACS patients. Although age ≥ 70 years was a predictor of one-year mortality, the mortality rates in the two groups were similar.

Thrombolysis was performed in a minority of patients despite a lack of contraindications. This may reflect the availability of urgent angiography at most of the enrolling centres. Almost all patients were referred for angiography with high intervention rates. There was a preference for the use of drug-eluting stents as opposed to bare metal stents. Revascularisation rates were high and certainly higher than the overall ACCESS data (68.5 vs 40.8%), but were similar for NSTE-ACS and STEMI (68.75 and 68.6%) patients. Therefore, approximately one-third of all patients were being treated conservatively/medically only.

The use of appropriate ancillary drug therapy in hospital and on discharge was in line with other registries; more than 90% receiving a statin, 70% a beta-blocker and 70% some form of RAAS blockade. As to be expected, the use of calcium channel blockers was low (13%).

Use of anti-thrombotic therapy was also in keeping with guidelines; 94% receiving aspirin, two-thirds clopidogrel, and 65% heparin (with LMWH being used more commonly). However, the use of a thienopyridine at discharge was lower in the South African cohort (62.2%) compared with the ACCESS population (76.1%), despite a higher intervention rate and usage of stents, and in particular, drug-eluting stents. One has to assume that patients receiving a stent were discharged on a thienopyridine.

This therefore leaves the suggestion that patients being referred for surgery or undergoing angiography without a PCI are not receiving a thienopyridine at discharge despite the diagnosis of an acute coronary syndrome. This is particularly worrying for those patients without STEMI, in whom there are clear guidelines for the prescription of a thienopyridine.^10^

## ACS registry populations

In terms of ACS types, patients enrolled in the ACCESS South Africa study were broadly similar to those in other observational studies conducted in Western populations. Just over half (56%) of the patients were diagnosed with NSTE-ACS and 39% with STEMI. In the second Euro-Heart Survey of Acute Coronary Syndromes (EHS-ACS-II) conducted in 2004, which included 6 385 patients from 32 European countries, the ACS population comprised almost equal proportions with NSTE-ACS and STEMI (48 and 47%, respectively) patients.^11^ In the GRACE study, which enrolled patients in 14 countries in Europe, North and South America, and Australia and New Zealand between 1999 and 2007,^12^-^14^ the proportion of patients with STEMI ranged from 30–40%, and 60–70% were diagnosed with NSTE-ACS.

Patients enrolled in ACCESS South Africa were younger than those enrolled in other Western observational studies (58 vs 63–66 years),^11^,^15^ and there were more men (75 vs 64–72%).^11^,^15^,^16^ Current smoking (43.9%) and diabetes were also common (23.9%). The actual prevalence of diabetes may have been even higher, as the diagnosis was based on a prior history and did not include admission hyperglycaemia and the subsequent diagnosis of diabetes.

When compared with the CREATE registry conducted exclusively in India and involving over 20 000 patients with an ACS, ACCESS South Africa had a higher percentage of patients aged 70 years and older (17.7 vs 13%), an equivalent percentage of male patients (75 vs 76%), and a much lower percentage with STEMI (39.4 vs 61%).^17^

## Limitations

The ACCESS South Africa study was an observational study and had all the limitations that such a study inherently has. The enrolling centres were all located in urban areas and were largely those that provided tertiary care (i.e. they had facilities for angiography and interventional therapy). Almost all enrolling centres were from the private healthcare sector. Patients were therefore mostly those that had access to private healthcare funding. These factors may all have influenced the population of patients studied.

Patients enrolled had to be alive upon admission and provide consent. This therefore excluded ACS patients who died prior to hospitalisation or within a short time of arrival. It therefore represents more the demographics of survivors of the acute phase of ACS. As in all observational studies, the data were non-randomised and treatment protocols were non-standardised.

Although the study called for recruitment of consecutive patients, it cannot be verified to what extend this aspect was complied with, and hence patient selection bias may also have been present. The number of patients lost to follow up (5.2%) is always of concern. However this was less than that of the overall ACCESS registry data (8.4%) and is probably acceptable for a registry, as opposed to a trial.

The data for the registry were compiled between January 2007 and January 2008. Subsequent to this, guidelines have changed and with this, changes in treatment protocols and prescription habits may also have occurred. Therefore some data may be out dated, but are nevertheless still relevant.

## Conclusions

In this study of ACS patients in South Africa, management tended to be aggressive, with high percentages of patients receiving angiography and an invasive treatment strategy. Drug usage was comparable to that in other countries and registries. Although this invasive approach may seem to be potentially costly, the 12-month death rate compared very favourably with other observational reports, and re-admission rates for bleeding and recurrent ischaemic events were low, suggesting that the cost may be justified and even economical in the long term.

The use of evidence-based medications and interventions is in line with practices in the developed world. There is however still room for improvement. The drop-off in drug usage over time is of concern, particularly the finding that only 77% of patients were still on a statin at one year. Also the discharge prescription of thienopyridines was relatively low, particularly given the nature of the population studied. Increased efforts need to be directed at proven secondary prevention treatment strategies.

This study was by no means complete or exhaustive, but serves as a template for practitioners and healthcare funders to take cognisance of the findings, make amendments to existing treatment protocols, and endeavour to improve drug utilisation and ultimately patient outcomes.
